# Recombinant Phytase Modulates Blood Amino Acids and Proteomics Profiles in Pigs Fed with Low-Protein, -Calcium, and -Phosphorous Diets

**DOI:** 10.3390/ijms25010341

**Published:** 2023-12-26

**Authors:** Cedrick N. Shili, Frank Kiyimba, Steve Hartsen, Ranjith Ramanathan, Adel Pezeshki

**Affiliations:** 1 Department of Animal and Food Sciences, Oklahoma State University, Stillwater, OK 74078, USA; cshili@alcorn.edu (C.N.S.); frank.kiyimba@okstate.edu (F.K.); ranjith.ramanathan@okstate.edu (R.R.); 2 Department of Biochemistry and Molecular Biology, Oklahoma State University, Stillwater, OK 74078, USA; steven.hartson@okstate.edu

**Keywords:** low-protein diet, corn-expressed phytase, proteomics

## Abstract

A beneficial effect of corn-expressed phytase (CEP) on the growth performance of pigs fed with very low-protein (VLP) diets was previously shown. Little is known whether this improvement is related to alterations in the expression profiles of blood proteins and amino acids (AAs). The objective of this study was to investigate whether supplementation of VLP, low-calcium (Ca), and low-P diets with a CEP would alter the blood AAs and protein expression profiles in pigs. Forty-eight pigs were subjected to one of the following groups (*n* = 8/group) for 4 weeks: positive control (PC), negative control-reduced protein (NC), NC + low-dose CEP (LD), NC + high-dose CEP (HD), LD with reduced Ca/P (LDR), and HD with reduced Ca/P (HDR). Plasma leucine and phenylalanine concentrations were reduced in NC; however, the LD diet recovered the concentration of these AAs. Serum proteomics analysis revealed that proteins involved with growth regulation, such as selenoprotein P were upregulated while the IGF-binding proteins family proteins were differentially expressed in CEP-supplemented groups. Furthermore, a positive correlation was detected between growth and abundance of proteins involved in bone mineralization and muscle structure development. Taken together, CEP improved the blood profile of some essential AAs and affected the expression of proteins involved in the regulation of growth.

## 1. Introduction

Very low-protein (VLP) diets with more than 25% reduced crude protein than recommended levels for swine [1] could be beneficial for decreasing nutrient excretion and the incidence of diarrhea in pigs [2,3,4]. However, these diets, while supplemented with the first four limiting amino acids (AAs, i.e., lysine, methionine, tryptophan, and threonine) decrease the feed efficiency and growth performance of pigs at different stages of life [4,5,6,7,8]. Further research is needed to develop effective and novel strategies to improve the growth performance of pigs fed with VLP diets so that they can be used in commercial swine production.

Microbial phytase supplemented to diets improve the digestibility of AAs and protein utilization in pigs [9,10,11,12,13,14]. Therefore, phytase may have the potential to reduce the need for high-protein diets by improving the efficiency of protein utilization. Unlike microbial phytase, which is produced during the fermentation process, recombinant phytase is produced through its expression in transgenic plants. Recombinant phytase can be an economical alternative to microbial phytase due to the fact that a large biomass of plants can express the transferred genes of phytase at a larger scale [15]. Supplementing normal protein, but low-phosphorus (P) diets with a corn-expressed phytase (CEP) was shown to improve average daily gain, P digestibility, and bone characteristics in nursery [16] and growing pigs [17]. Importantly, we showed that supplementation of VLP diets with CEP, regardless of the doses used (i.e., 2000 and 4000 FTU/kg), improved the average daily gain (ADG) and gain:protein (G:P) of weaned pigs [18].

In our previous study, we showed that the positive effect of CEP on the growth performance of pigs fed with VLP diets was associated with increased digestibility of calcium (Ca) and P, bone characteristics, and differential fecal bacterial population [18]. There is some evidence that microbial phytase may improve the blood AAs profile in broilers [19,20]. This improvement in the profile of AAs has been shown to be linked with enhanced growth performance in pigs [21]. Likewise, serum insulin-like growth factors (IGFs) and IGF-binding proteins (IGFBPs) are known for their regulatory role in growth, development, differentiation, and other physiological activities [22]. The serum proteome profile in pigs has been previously reported [23,24], but no data are available on alterations in protein expression profiles when phytase is supplemented in the diet. Little is known about whether the profile of blood proteins and other nitrogen-containing compounds, such as AAs, are also related with enhanced growth performance of pigs fed with VLP diets supplemented with CEP. Thus, the objective of this study was to investigate the effect of a CEP on blood metabolites, nitrogen-containing compounds, serum protein expression profiles, and body composition in pigs fed with VLP diets.

## 2. Results

### 2.1. Plasma Metabolites and Body Composition

Pigs fed with HD had 119% (*p* ≤ 0.05) and 97% (*p* ≤ 0.1) higher plasma triglycerides than those fed with NC and LD diets, respectively (Table 1). No differences were detected among groups on plasma concentration of glucose and cholesterol. Pigs fed with VLP diets (i.e., NC, LD, HD, LDR, and HDR) had lower body lean mass than PC. Relative to the PC group, pigs in the LD, HD, and HDR groups had lower body lean percent, but NC and LDR did not differ. Relative to PC, pigs fed with NC, HD, and LDR had a lower body fat mass, but LD and HDR did not change. Further, pigs fed with LD and HDR had 38% and 43% higher fat mass than those fed with the NC diet. Pigs fed with the LD and HDR diets had 20% and 23% higher body fat percentages compared to those fed with a PC diet, respectively.

### 2.2. Plasma Nitrogen-Containing Compounds

Pigs fed with VLP diets (i.e., NC, LD, HD, LDR, and HDR) had a lower plasma concentration of isoleucine, valine, histidine, arginine, tyrosine, and asparagine and a higher plasma concentration of threonine, methionine, and lysine than PC (Table 2). Compared to PC, the NC diet reduced the plasma leucine and phenylalanine concentrations; however, LD recovered their levels. Additionally, pigs fed with HD had a higher plasma alanine and glycine and tended to have a greater serine than PC. The rest of the plasma AAs did not show significant or meaningful changes across dietary groups (Table 2).

### 2.3. Differentially Abundant Proteins in Serum of Pigs 

**Figure 1:**
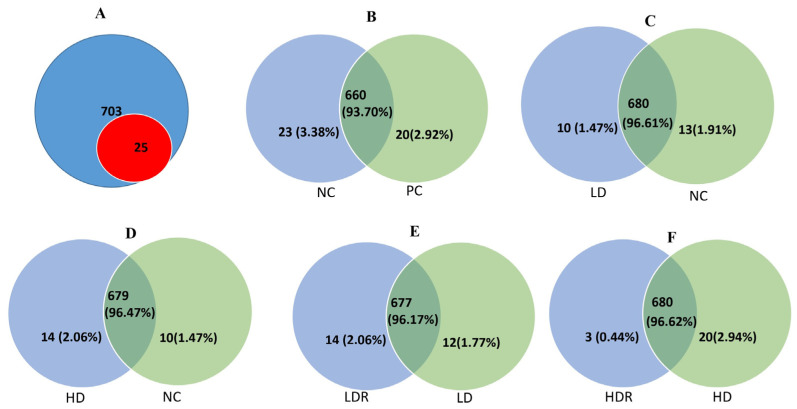
Venn diagrams of total identified proteins in the serum of pigs fed with low-protein diets with or without reduced calcium and phosphorous supplemented with a corn-expressed phytase. (**A**) the number of total identified proteins across biological replicates (703 proteins) with 25 proteins identified with significantly differential abundance across dietary groups, (**B**) NC vs. PC, (**C**) LD vs. NC, (**D**) HD vs. NC, (**E**) LDR vs. LD, (**F**) HDR vs. HD. PC (positive control): normal protein, adequate calcium (Ca) and available phosphorous (aP), no corn-expressed phytase (CEP) added; NC (negative control): low protein, adequate Ca and aP, no CEP added; LD: NC + CEP added at low dose, i.e., 2000 one phytase unit (FTU)/kg of diet; HD: NC + CEP added at high dose, i.e., 4000 FTU/kg of diet; LDR: LD with reduced Ca and P; HDR: HD with reduced Ca and P. *n* = 7–8 for each dietary group.

Following serum proteomics analysis, 703 proteins were identified and quantified in the serum proteome of pigs. Out of these, 25 proteins showed differential expression (*p* < 0.05) among all treatments (Figure 1A, and Table 3). When the NC was compared to the PC group, a total of 660 common proteins were present in both NC and PC groups (93.7%), of which 23 (3.38%) proteins were upregulated in the NC group and 20 (2.92%) proteins were upregulated in the PC group (Figure 1B). When LD and NC groups were compared, a total of 680 common proteins were present in both groups (96.61%); of these, 10 (1.47%) proteins were upregulated in the LD group and 13 (1.91%) proteins were upregulated in the NC group (Figure 1C). The comparison between HD and NC revealed a total of 679 common proteins (96.47%) being present in both groups; of these, 14 (2.06%) proteins were upregulated in the HD group, and 10 (1.45%) proteins were upregulated in the NC group (Figure 1D). While the comparison between the LDR and LD groups showed a total of 677 common proteins (96.17%), 14 (2.06%) of proteins were upregulated in LDR and 12 (1.77%) proteins were upregulated in LD (Figure 1E). Furthermore, when HDR vs. HD groups were compared, a total of 680 common proteins were present in both HDR and HD groups (96.62%); of these, three (0.44%) proteins were upregulated in HDR, and 20 (2.94%) proteins were upregulated in the HD group (Figure 1F). 

**Figure 2:**
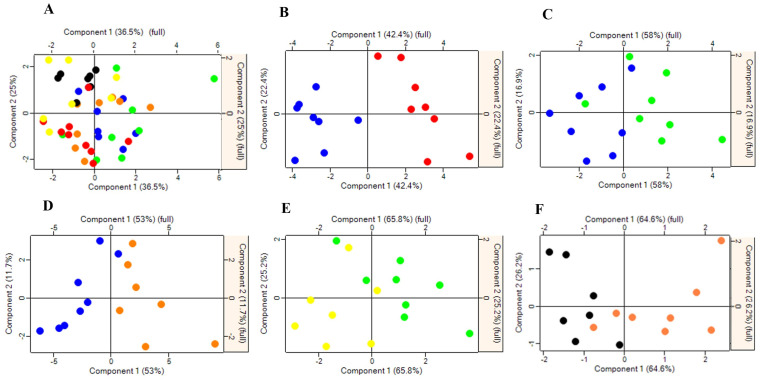
Principal Component Analysis (PCA) score plots of differentially expressed protein in the serum of pigs fed with low-protein diets with or without reduced calcium and phosphorous supplemented with a corn-expressed phytase. (**A**) Overall, (**B**) NC vs. PC, (**C**) LD vs. NC, (**D**) HD vs. NC, (**E**) LDR vs. LD, (**F**) HDR vs. HD. Each circle represents an individual pig and the red, blue, green, orange, yellow and black circles are representative of PC, NC, LD, HD, LDR and HDR groups, respectively. PC (positive control): normal protein, adequate calcium (Ca) and available phosphorous (aP), no corn-expressed phytase (CEP) added; NC (negative control): low protein, adequate Ca and aP, no CEP added; LD: NC + CEP added at low dose, i.e., 2000 one phytase unit (FTU)/kg of diet; HD: NC + CEP added at high dose, i.e., 4000 FTU/kg of diet; LDR: LD with reduced Ca and P; HDR: HD with reduced Ca and P. *n* = 7–8 for each dietary group.

**Figure 3:**
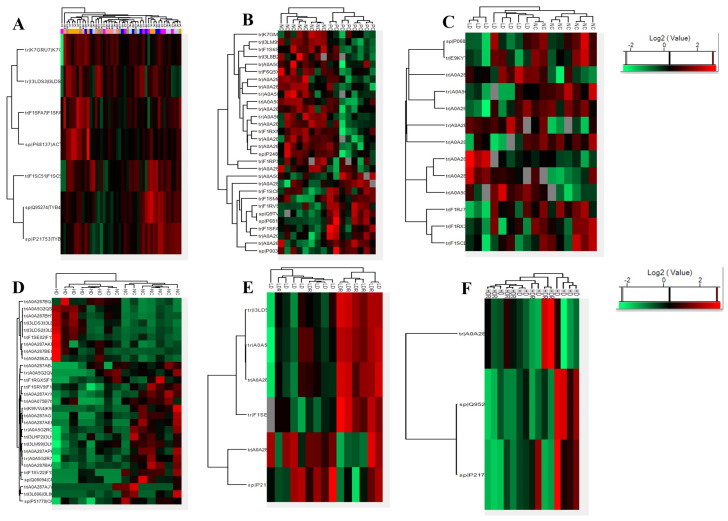
Hierarchical clustering of differentially expressed protein in serum of pigs fed with low-protein diets with or without reduced calcium and phosphorous supplemented with a corn-expressed phytase. (**A**) Overall, (**B**) NC vs. PC, (**C**) LD vs. NC, (**D**) HD vs. NC, (**E**) LDR vs. LD, (**F**) HDR vs. HD. The red color indicates high abundance, and the green color indicates low abundance. PC (positive control): normal protein, adequate calcium (Ca) and available phosphorous (aP), no corn-expressed phytase (CEP) added; NC (negative control): low protein, adequate Ca and aP, no CEP added; LD: NC + CEP added at low dose, i.e., 2000 one phytase unit (FTU)/kg of diet; HD: NC + CEP added at high dose, i.e., 4000 FTU/kg of diet; LDR: LD with reduced Ca and P; HDR: HD with reduced Ca and P. *n* = 7–8 for each dietary group.

To better assess how phytase supplementation might influence changes in growth performance and serum protein expression profiles, a pairwise comparison was employed. The principal component analysis (PCA) showed a distinct separation between different dietary treatments (Figure 2A–F), suggesting that each treatment group exhibits differential protein expression profiles when compared to their control groups. Furthermore, hierarchical clustering analysis showed distinctive clusters of upregulated and downregulated proteins among groups (Figure 3A–F). These data show that the protein expression profile of each dietary group was unique.

To further explore the functional annotations of the differentially abundant proteins within clusters, Gene Ontology (GO) enrichment analysis was employed. GO results showed enrichment for processes that are implicated in the regulation of growth factor activity and developmental processes, including connective tissue development and muscle system process (Figures S1–S4). Compared to PC, NC upregulated a group of proteins, such as ameloblastin (AMBN) and insulin-like growth factor-binding protein 2 (IGFBP2), that are involved in the regulation of growth factor activity and IGF-1 binding molecular functions, respectively (Table S1). Relative to PC, NC downregulated a group of proteins, including collagen type I alpha 2 chain (COL1A2), IGF-1, and peptidase inhibitor 16 (PI16), which were involved in bone mineralization, growth regulation and regulation of cell growth involved in cardiac muscle cell development, respectively (Table S1). Compared to NC, LD upregulated selenoprotein P (SELENOP) and collagen type V alpha 1 (COL5A1) that are involved in the regulation of growth and blood vessel development, respectively. Relative to NC, LD downregulated IGFBP4 and biglycans (BGN) that control growth regulation by IGFs and collagen fiber assembly, respectively. In the HD vs. NC comparison, pigs fed with HD diets upregulated IGFBP3 and secretogranin V (SCG5) that are involved in the regulation of IGF receptor signaling pathway and of hormone secretion, respectively, while they downregulated GC vitamin D binding protein (GC) and vasorin (VASN) that are part of vitamin D metabolic process and cellular response to redox state, respectively (Table S1). In the LDR vs. LD comparison, LDR upregulated TMF1 regulated nuclear protein 1 (TRNP1), arginase 1 (ARG1), heat shock protein family E (HSPE1) and biglycans (BGN) that are important for regulation of cell cycle involved in urea cycle, protein folding and collagen fiber assembly, respectively. Relative to LD, LDR downregulated grancalcin (GCA), lipocalin 15 (LCN15) and lecithin-cholesterol acyltransferas (LCAT) that play an important role in calcium ion binding, retinol binding and cholesterol metabolic process, respectively. Compared to HD, HDR upregulated myristoylated alanine-rich protein kinase C substrate (MARCKS) involved in actin crosslink formation, while it downregulated thymosin beta 4 X-linked (TMSB4) and calpain small subunit 1 (CAPNS) involved in the regulation of cell migration and proteolysis, respectively.

**Figure 4:**
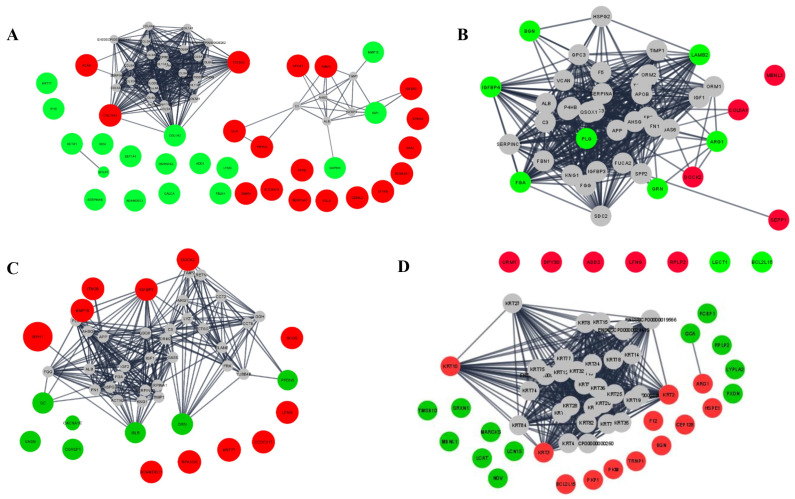
Network analysis of differentially expressed proteins in serum of nursery pigs fed with low-protein diets with or without reduced calcium and phosphorous supplemented with a corn-expressed phytase. Network of interactions of differentially expressed proteins for (**A**) NC vs. PC, (**B**) LD vs. NC, (**C**) HD vs. NC, (**D**) LDR vs. LD. No protein interactions were detected for HDR vs. HD. The red and green colors are representative of upregulated and downregulated proteins, respectively, and the grey color represents 40% of additional interactors. PC (positive control): normal protein, adequate calcium (Ca) and available phosphorous (aP), no corn-expressed phytase (CEP) added; NC (negative control): low protein, adequate Ca and aP, no CEP added; LD: NC + CEP added at low dose, i.e., 2000 one phytase unit (FTU)/kg of diet; HD: NC + CEP added at high dose, i.e., 4000 FTU/kg of diet; LDR: LD with reduced Ca and P; HDR: HD with reduced Ca and P. *n* = 7–8 for each dietary group.

To further understand the potential protein–protein interaction between the upregulated and downregulated proteins among the different treatments group, a network analysis was employed. When comparing the differentially abundant proteins between PC and NC groups (Figure 4A), few connections were found between the proteins regulating growth (i.e., IGF-1, IGFBP2). When LD vs. NC groups were considered (Figure 4B) network analysis revealed an interaction between the upregulated proteins (DOCK2), and the downregulated proteins (ARG1), and between the downregulated proteins such as LAMB2 and BGN, and IGFBP4, FGA, and PLG. These proteins are involved in platelet-derived growth factor binding, guanyl-nucleotide exchange factor activity, IGF-I binding, and urea cycle molecular functions. Comparing HD vs. NC (Figure 4C), the upregulated proteins (IGFBP3, DOCK2), showed interactions with the downregulated proteins (DRN, PFDN5, GC, CACNA1E). The LDR vs. LD comparison (Figure 4D) showed only connections between three upregulated proteins (i.e., KRT10, KRT3 and KRT2). 

**Figure 5:**
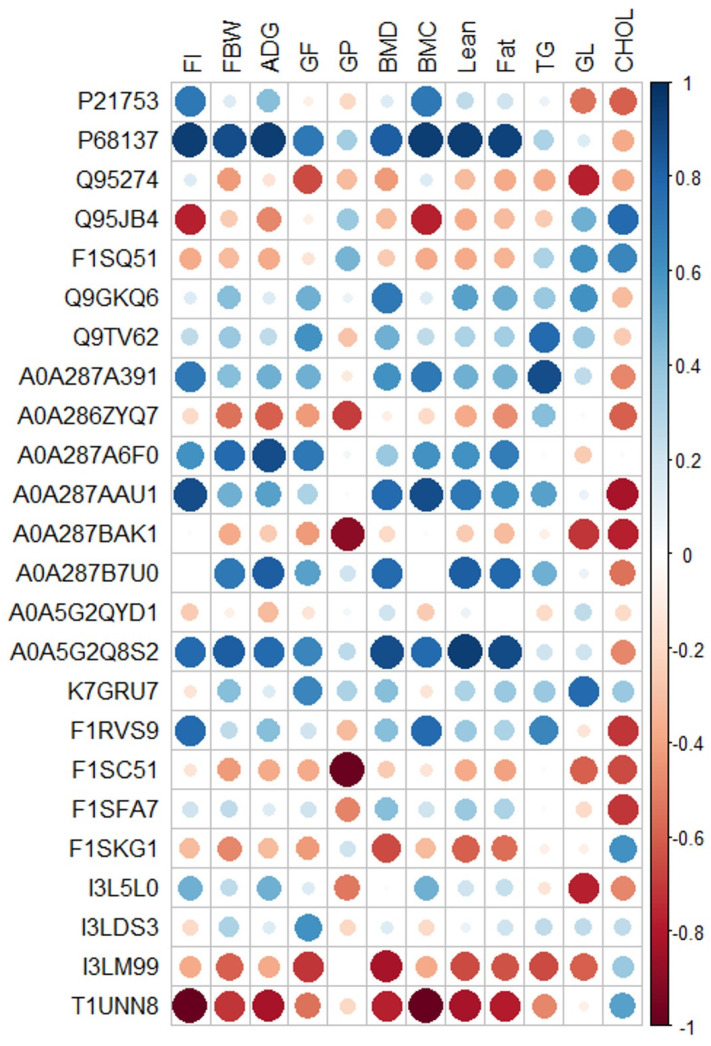
Pearson correlation analysis of overall differentially expressed proteins (protein ID) in serum of pigs fed with low-protein diets with or without reduced calcium and phosphorous supplemented with a corn-expressed phytase identified by proteomics approach and growth performance related measurements. The color of circles is based on the Pearson coefficient distribution: red represents a negative correlation (*p* < 0.05), blue represents a positive correlation (*p* < 0.05), and white is indicative of nonsignificant correlation (*p* > 0.05) with larger size circles having a higher correlation coefficient. Feed intake (FI), final body weight (FBW), average daily gain (ADG), gain:feed ratio (G:F), gain:protein ratio (GP), bone mineral density (BMD), bone mineral content (BMC), triglyceride (TG), glucose (GL), cholesterol (CHOL).

To examine the relationship between the serum proteomics profile and the growth performance of pigs supplemented with CEP from our previously published data [18], the Pearson correlation analysis was employed (Figure 5). The results showed that there is a significant positive correlation between feed intake (FI), ADG, final body weight (FBW), G:F ratio, bone mineral density (BMD), bone mineral content (BMC), body lean, and fat mass with changes in serum protein expression of P68137 (ACTA1), A0A287A6F0 (MYLPF), A0A287A391 (EEF1A1), A0A287AAU1 (CCN3), A0A287B7U0 (RPLP2), A0A5G2Q8S2 (PXDN), and K7GRU7 (COL1A2). Also, there was a negative correlation between FBW, G:F ratio, BMD, BMC, body lean and fat mass with the changes in expression of Q95274 (TMSB4), Q95JB4 (LEAP2), A0A286ZYQ7 (IGKV2D-40), A0A287BAK1 (TLN1), F1SKG1 (INHBE), I3LM99 (GCA), and T1UNN8 (ANGPTL8). 

## 3. Discussion

Very low-protein diets with reduced Ca, and P could potentially be used to reduce the environmental concerns associated with the excretion of nutrients, post-weaning diarrhea, and feed costs in pigs [2,3,4]. However, these diets have a negative influence on the growth performance and health of pigs [7,8]. We previously showed that supplementation of VLP diets with CEP improved the growth performance of weaned pigs [18]. The objective of the present study was to investigate whether the profile of blood proteins and other nitrogen-containing compounds, such as AAs, are related to enhanced growth performance of pigs fed with VLP diets supplemented with CEP. Here, we employed a proteomics approach to study the protein distribution in major body fluid, serum. Proteomics could be used to improve animal production and agricultural sustainability by identifying key biomarkers of growth. Our study revealed several important findings: (1) a very low-protein diet reduced the plasma leucine and phenylalanine concentrations, but the LD diet recovered the concentration of these two AAs. Pigs fed with the HD diet had a higher plasma alanine, glycine, and serine than PC; (2) the LD diet changed the expression of serum proteins such as SELENOP, COL5A1, IGFBP4, and BGN that are involved in the regulation of growth and blood vessel development, while the HD diet altered the expression of proteins such as IGFBP3, SCG5, GC, and VASN that are involved in regulation of IGF receptor signaling pathway, hormone secretion, vitamin D metabolic process, and cellular response to redox state; and (3) A positive correlation was found between growth performance parameters, BMD, BMC, and body composition with changes in expression of proteins such as ACTA1, MYLPF, EEF1A1, CCN3, RPLP2, PXDN, and COL1A2, but there was a negative correlation between these parameters with the changes in expression of proteins such as TMSB4, LEAP2, IGKV2D-40, TLN1, INHBE, GCA, and ANGPTL8. Overall, CEP improved the blood profile of some essential AAs and affected the expression of proteins involved in the regulation of growth and hormone secretion and metabolic processes in pigs fed with VLP diets.

A very low-protein diet reduced the plasma glycine but feeding pigs with the HD diet recovered the reduced level of glycine. It has been reported that supplementing diets with phytase enhances the AAs and total alpha-amino N digestibility in pigs and broilers fed with standard protein diets [13,25,26]. Similarly, others showed that feeding pigs with standard protein diets supplemented with microbial phytase raised the plasma concentrations of nitrogen-containing compounds a few hours following a meal in finishing pigs [27]. Authors indicated that the addition of phytase improved the absorption of AAs [27]. Also, Lala et al., (2020) reported an improvement in the ileal digestibility of lysine, threonine, and tryptophan in growing pigs fed with a low-protein (14% CP) diet when phytase was supplemented [28]. This improvement in the profile of AAs is linked with enhanced growth performance in pigs [21]. Our data suggest that CEP recovers the decreased concentration of some plasma AAs in pigs fed with VLP diets. Further research is needed to better understand the role of CEP on the expression of AA transporters in the gut and other peripheral tissues, such as the skeletal muscle of pigs fed with VLP diets.

Supplementation of VLP diets with CEP did not change the concentration of plasma glucose, cholesterol, and triglycerides when compared to the PC diet. These results are in general agreement with previous studies showing no effect of phytase supplementation on blood metabolites in broilers [29,30]. These data suggest that CEP has no adverse effects on plasma metabolites and likely liver function. In future studies, assessing the hematological parameters, liver enzymes and other biochemical parameters in plasma following CEP supplementation will provide better insights into the possible impacts of CEP on immune function, metabolism, and health of pigs.

Although serum protein expression profiles of pigs have been previously reported [23,24,31], no data are available on alterations in serum proteomic profile when CEP is supplemented to VLP diets in pigs. In the current study, we found that supplementing VLP diets with CEP, regardless of the doses used, changed the serum protein expression profiles. Several proteins involved in growth regulation, such as IGFBP3 and IGFBP4, showed differential abundance in LD, LDR, HD, and HDR groups suggesting an important role of these proteins in mediating the positive effects of CEP on growth performance. The IGFBPs have been shown to transport IGFs in circulation and deliver them to specific tissues, thereby modulating the bioavailability of IGFs to their receptors [32]. This important role in the GH (GH)-IGF-1 axis promotes skeletal growth through the regulation of cellular differentiation and proliferation [33]. There is enough evidence suggesting that GH is a major regulator of linear bone growth. Endocrine actions of GH are via hepatic IGF-1 production, whereas local (autocrine/paracrine) actions may be direct or IGF-1-mediated [34]. Additionally, IGF-1 facilitates protein synthesis in muscle by activating mTOR signaling pathways [35]. Thus, the greater abundance of IGF-like proteins, especially in the HD groups could, in part, explain the improvement in growth performance observed in pigs supplemented with CEP. 

The concentration of IGF-1 decreased when pigs were fed with VLP diets in the current study. Likewise, we [36] and others [37,38] previously showed that the concentration of serum IGF-1 is decreased in pigs fed with VLP diets. More recently, we showed that the mRNA abundance of hepatic IGF-1 was lower in pigs receiving VLP diets [39]. The reduced concentration of circulating IGF-1 in VLP pigs could be due to a lower concentration of serum-branched chain amino acids (i.e., Leu, Ile, and Val) in these pigs, as seen in this study and our previous works [36,40,41]. There is evidence that Leu induces the secretion of IGF-1 [42]. IGF-1 is involved in nutritional regulation of growth [43]. Unlike IGF-1, the concentration of serum IGFBP2 increased in VLP pigs. IGFBP2 is considered a negative regulator of growth via reducing the IGF-1 bioavailability [44]. A lower concentration of serum IGF-1 and a higher concentration of IGFBP2 may contribute to the adverse effects of VLP diets on growth performance. 

Supplementation of CEP differentially changed the profile of several proteins involved in growth regulation. LD increased serum SELENOP concentration, which reflects the total selenium status of the whole body [45,46]. SELENOP-knockout mice display a phenotype with growth deficits suggestive of an important role of selenium and SELENOP in growth regulation [47]. Further, LD reduced the serum IGFBP4. It is known that IGFBP 4 has different endocrine, autocrine, and paracrine roles in the regulation of bone growth. In one study, overexpression of osteoblast-specific IGFBP4 reduced bone turnover and growth [48]. Supplementing a higher dose of CEP (i.e., HD) increased the serum concentration if IGBP3. IGFBP3, also known as somatomedin C, plays an important role in cellular growth, differentiation, and development through the delivery of IGFs to target cells [49]. Increased serum concentration of SELENOP and IGFBP3 while decreased IGFBP4 likely contributes to improved growth performance following supplementation of CEP.

Overall, new emerging technologies have come to play an important role in the challenges facing the sustainable agriculture industry. In particular, proteomics may potentially contribute significantly to the health and production of animals by identifying candidate molecules that are responsible for feed efficiency and efficient growth. Further, omics technologies such as proteomics can be combined with other molecular and breeding methods used by industry to improve productivity in the animal production industry. Overall, omics provide remarkable opportunities to better understand animal biology and identify biomarkers underlying important economic traits and diseases.

## 4. Materials and Methods

### 4.1. Animals and Experimental Design

The experimental procedures used in this study were according to the Federation of Animal Science Societies (FASS) Guide for the Care and Use of Agricultural Animals in Research and Teaching (Ag Guide) [50]. All the experimental procedures were approved by the Oklahoma State University’s Institutional Animal Care and Use Committee (Animal Care and Use Protocol # AG-17-22). The details of the animal experiments and diets were described previously [18]. Briefly, following 2 weeks of acclimation period, 48 weaned barrows were weight-matched, housed individually and randomly allotted to six dietary treatments (*n* = 8/treatment) for 4 weeks: (1) positive control with normal protein content (PC); (2) negative control with low-protein content (NC); (3) NC + low dose of CEP (2000 FTU/kg, LD); (4) NC + high dose of CEP (4000 FTU/kg, HD); (5) LD with reduced Ca/P (LDR); and (6) HD with reduced Ca/P (HDR) [18]. At the end of the study (week 6), blood samples were collected and processed, and serum and plasma were stored at −80 °C. The pigs were euthanized and scanned by dual-energy X-ray absorptiometry (DEXA), as we previously described [18]. 

### 4.2. Serum Triglycerides, Glucose, and Cholesterol

Serum triglycerides, glucose, and cholesterol were analyzed using an automated chemistry analyzer system (CLC 480/BioLis24i, Carolina Liquid Chemistries Corp., Brea, CA, USA) as previously described [36,51]. The reagents for triglycerides (Catalogue #: BL213), glucose (Catalogue #: BL208), and cholesterol (Catalogue #: BL211) were obtained from Carolina Liquid Chemistries Corp. (Brea, CA, USA). The triglyceride and cholesterol absorbance were detected at 505 nm, and glucose absorbance was detected at 340 nm. 

### 4.3. Plasma Nitrogen-Containing Compounds Analysis 

Plasma samples were analyzed for AAs and other nitrogenous-containing compounds at the Molecular Structure Facility, Proteomics Core (UC Davis Genome Center, Davis, CA, USA), as we previously described [52]. Briefly, samples were acidified with 2% sulfosalicylic acid and incubated at 25 °C for 15 min. Following overnight storage of samples at −20 °C, prior to injection (50 µL), samples were diluted with Li sample diluent (Pickering Labs, Mountain View, CA, USA). An ion-exchange chromatography method using HITACHI L-8900 Amino Acid Analyzer (Hitachi High-Technologies Corporation, Tokyo, Japan) with a post-column ninhydrin reaction was applied to separate free AAs. For the calibration of the AA analyzer, the AA standards (Sigma-Aldrich, St. Louis, MO, USA) were utilized. To determine the response factor for each AA and AA concentration relative to the known ones, the related compounds’ standards absorbance was measured at both 570 nm and 440 nm following the reaction with ninhydrin. To consider variations in injection volume, the internal standard (AE-Cys, Sigma #A2636) was also included.

### 4.4. Serum Proteomics Analysis

To deplete abundant proteins with large molecular weight, such as albumins and immunoglobulins from serum, samples were processed by the “depth filtration” approach as previously described [53,54]. For this, 50 µL of serum was mixed with 75 µL of 100% acetonitrile, and the reaction was incubated at room temperature for 30 min. After incubation, tubes were centrifuged at 10,000× *g* for 30 min at 22 °C, the supernatant was collected and loaded into the top segment of a commercial 200 µL pipet tip packed with monolithic C18 (ThermoFisher, Waltham, MA, USA), which was then attached to a pipettor to force the samples through the packed bed. These filtrates were then dried in a vacuum centrifuge and redissolved for 30 min in buffered 8M urea containing 5 mM tris (2-carboxyethyl) phosphine. After dissolution and reduction, samples were alkylated with 10 mM iodoacetamide for 20 min, diluted with 3 volumes of 100 mM Tris-HCl (pH 8.2), and digested at 37 °C with 1 µg of trypin/LysC (Promega Madison, WI, USA). After overnight digestion, an additional 0.5 µg aliquot of protease was added, and the reactions were incubated for an additional 6 h. Digestions were desalted by solid phase extraction using monolithic C18 pipet tips (ThermoFisher, Waltham, MA, USA), dried by vacuum centrifugation, and analyzed by LC-MS/MS.

For LC-MS/MS analyses, samples were dissolved in 45 µL of mobile phase A (0.1% aqueous formic acid), diluted further with 2.4 volumes of mobile phase A, and 10 µL of peptides were injected into a 75 µm × 50 cm C18 analytical column using a trap column configuration (Thermo PN 164942 and PN 164535, resp.). Peptides were then eluted using a gradient of 2.5–30% acidified acetonitrile delivered at 250 nL/min over a 120 min HPLC run. Eluting peptides were ionized in a Nanospray Flex ion source using stainless-steel emitters and analyzed in a Quadrupole-Orbitrap “Fusion” mass spectrometer (Thermo Waltham, MA, USA). The peptide ion stream was analyzed using a “top speed, high/low” MS/MS method that utilized the Orbitrap sector to detect peptide parent ions, the quadrupole sector for data-dependent ion selection for MS/MS, the ion routing multipole sector for MS/MS fragmentation by HCD, and the ion trap sector to analyze MS/MS fragments.

Data were analyzed by using MaxQuant v1.6.10.43 [55] to search the instrument RAW files, concatenating four RAW files as “fractions” for each sample. Searches used the default MaxQuant parameters, supplemented with the variable modification Gln cyclization to pyro-glutamate, use of the match-between-runs feature to transfer MS/MS identifications between MS peaks, and use of the label-free quantification (LFQ) algorithm [55,56] for protein quantitation. LFQ protein intensities were analyzed using Perseus.

### 4.5. Bioinformatics Analysis of the Differentially Abundant Proteins 

The proteomics data were analyzed by several approaches. MaxQuant (v1.5.2.8) was employed for protein identification and normalization of LFQ MS/MS data. MS/MS data were searched against a Uniprot reference proteome database of Sus scrofa using default MaxQuant settings with the addition of match between runs. All statistical analyses, processing and visualization were performed using Perseus (V1.6.2.1) biostatistics software. ANOVA-based multi-sample t-test were performed using a cut-off of *p* < 0.05 of log2-transformed LFQ protein intensities to identify statistically different abundant proteins for 5 treatment comparisons (NC vs. PC, LD vs. NC, HD vs. NC, LDR vs. LD, HDR vs. HD). 

Principal Component Analysis (PCA) plots and hierarchical clustering of differentially abundant proteins were constructed and visualized using Perseus (V1.6.2.1). Using David functional annotation tool, https://metascape.org/ (accessed on 25 February 2022), GO enrichment analysis was employed for the functional characterization of differentially abundant proteins into biological processes, molecular functions, and cell localization.

In addition, proteins with limited descriptions in the database were further explored via a literature search using Unipot, https://www.uniprot.org/uniprot/ (accessed on 21 March 2021). Further, protein–protein interaction network analysis was performed by querying the String database for known and predicted protein–protein interaction of the corresponding differentially abundant proteins up- and downregulated in the serum samples with a confidence score of 0.8 and 40 addition interactors. The obtained string protein–protein interactions were visualized in Cytoscape (V.3.8.0). 

### 4.6. Statistical Analysis 

The Shapiro–Wilk test of normality was used to test the normal distribution of data in SPSS (IBM SPSS Statistics Version 23, Armonk, NY, USA). The data for variables with non-normal distributions were then normalized using inverse distribution (IDF-normal) prior to statistical analysis. The concentration of serum metabolites, plasma nitrogen-containing compounds and body fat and lean content data were analyzed using the univariate ANOVA procedure of SPSS^®^ (IBM SPSS Statistics version 23, Armonk, NY, USA). Means of dietary groups were separated by Tukey’s post hoc analysis. *p* ≤ 0.05 and 0.05 < *p* ≤ 0.1 were considered as statistical significance and trends, respectively. For proteomics data, the pairwise student t-test was used to determine differently abundant proteins among specific groups of interest (NC vs. PC, LD vs. NC, HD vs. NC, LDR vs. LD, HDR vs. HD) in Perseus (V1.6.2.1) with differences being considered significant at *p* value ≤ 0.05.

## 5. Conclusions

In summary, supplementing VLP diets with CEP improved the blood profile of some essential amino acids and changed the abundance of serum proteins involved in the regulation of growth suggesting an important role of these proteins in mediating the effects of CEP on growth performance. Further, alterations in the expression of serum proteome that are important for bone mineralization and muscle structure development likely contributed to the beneficial effects of CEP on growth, body composition, and bone measurements in pigs fed with VLP diets.

## Figures and Tables

**Table 1:** Plasma metabolites and body composition of nursery pigs fed with low-protein diets with or without reduced calcium and phosphorous supplemented with a corn-expressed phytase.

Items	Diets ^1^						SEM ^2^	*p*-Value
	PC	NC	LD	HD	LDR	HDR		
Triglycerides, mg/dL	28.04 ± 3.94 ^ac^	15.75 ± 3.69 ^a^	17.65 ± 4.67 ^ab,^*	34.64 ± 3.94 ^bc,^*	26.26 ± 3.69 ^ab^	22.40 ± 4.26 ^ab^	1.83	0.02
Glucose, mg/dL	114.94 ± 11.3	105.32 ± 10.61	110.08 ± 11.3	130.34 ± 11.3	116.04 ± 11.3	146.24 ± 12.2	4.82	0.16
Cholesterol, mg/dL	74.97 ± 25.1	103.61 ± 23.5	128.60 ± 23.5	91.01 ± 25.1	162.46 ± 23.5	158.94 ± 23.5	10.46	0.07
Lean mass, kg	24.11 ± 0.86 ^a^	13.28 ± 0.81 ^b^	16.23 ± 0.81 ^b^	14.95 ± 0.81 ^b^	14.76 ± 0.86 ^b^	16.36 ± 1.02 ^b^	0.63	<0.01
Fat mass, kg	3.35 ± 0.19 ^ad^	2.03 ± 0.18 ^b^	2.82 ± 0.18 ^ac^	2.40 ± 0.18 ^bc^	2.40 ± 0.19 ^bc^	2.92 ± 0.23 ^cd^	0.10	<0.01
Lean, %	86.54 ± 0.49 ^a^	85.22 ± 0.46 ^ac^	83.58 ± 0.46 ^bc^	84.49 ± 0.46 ^bc^	84.93 ± 0.49 ^ac^	83.43 ± 0.58 ^bc^	0.24	<0.01
Fat, %	12.10 ± 0.50 ^a^	12.93 ± 0.46 ^ab^	14.50 ± 0.46 ^b^	13.56 ± 0.46 ^ab^	13.35 ± 0.50 ^ab^	14.87 ± 0.59 ^b^	0.23	<0.01

^1^ PC (positive control): normal protein, adequate calcium (Ca) and available phosphorous (aP), no corn-expressed phytase (CEP) added; NC (negative control): low protein, adequate Ca and aP, no CEP added; LD: NC + CEP added at low dose, i.e., 2000 one phytase unit (FTU)/kg of diet; HD: NC + CEP added at high dose, i.e., 4000 FTU/kg of diet; LDR: LD with reduced Ca and P; HDR: HD with reduced Ca and P. Values are means ± SE. *n* = 8 for each dietary group. ^2^ SEM: Standard error of mean. ^a–d^ Within a row, values without a common superscript letter differ (*p* ≤ 0.05). * Within a row, values with a common superscript symbol are tended to be different (0.05 < *p* ≤ 0.1).

**Table 2:** The composition (nmol/mL) of plasma nitrogen-containing compounds of nursery pigs fed with low-protein diets with or without reduced calcium and phosphorous supplemented with a corn-expressed phytase.

Item	Diets ^1^						SEM ^2^	*p*-Value
	PC	NC	LD	HD	LDR	HDR		
Valine	283.1 ± 36.5 ^a^	84.33 ± 30.8 ^b^	125.6 ± 33.3 ^b^	58.08 ± 30.8 ^b^	126.4 ± 30.8 ^b^	81.12 ± 30.8 ^b^	16.42	<0.01
Methionine	48.50 ± 10.6 ^a,^*	90.28 ± 10.6 ^ab,^*	111.3 ± 10.6 ^b^	109.0 ± 9.87 ^b^	106.7 ± 9.87 ^b^	116.5 ± 9.87 ^b^	5.35	<0.01
Threonine	586.7 ± 236 ^a^	1704 ± 219 ^b^	2243 ± 236 ^b^	1986 ± 219 ^b^	1766 ± 219 ^b^	2212 ± 236 ^b^	122.8	<0.01
Isoleucine	142.9 ± 21.2 ^a^	32.42 ± 17.9 ^b^	36.47 ± 19.6 ^b^	35.10 ± 17.9 ^b^	49.46 ± 17.9 ^b^	35.94 ± 17.9 ^b^	9.13	<0.01
Leucine	274.8 ± 19.3 ^a^	183.0 ± 16.3 ^b^	218.9 ± 17.6 ^ab^	185.9 ± 16.3 ^b^	183.6 ± 16.3 ^b^	186.7 ± 16.3 ^b^	8.13	<0.01
Phenylalanine	118.5 ± 7.9 ^a^	85.50 ± 6.7 ^bc^	100.0 ± 7.2 ^ac^	84.89 ± 6.7 ^bc^	96.82 ± 6.7 ^bc^	87.27 ± 6.7 ^bc^	3.20	0.02
Tryptophan	76.40 ± 6.94	83.18 ± 6.42	83.10 ± 6.94	85.85 ± 6.42	92.01 ± 6.42	96.04 ± 6.42	2.70	0.37
Lysine	212.9 ± 71.5 ^a^	716.1 ± 66.2 ^b^	847.1 ± 71.5 ^b^	807.7 ± 66.2 ^b^	779.7 ± 66.2 ^b^	793.9 ± 66.2 ^b^	42.26	<0.01
Histidine	120.7 ± 6.12 ^a^	58.80 ± 5.66 ^b^	65.71 ± 6.12 ^b^	52.67 ± 5.66 ^b^	56.86 ± 5.66 ^b^	56.12 ± 5.66 ^b^	4.27	<0.01
Arginine	243.1 ± 16.8 ^a^	89.72 ± 15.5 ^b^	85.50 ± 16.8 ^b^	96.85 ± 15.5 ^b^	67.77 ± 15.5 ^b^	56.20 ± 15.5 ^b^	11.40	<0.01
Aspartic acid	42.58 ± 8.29	43.74 ± 7.67	37.37 ± 8.29	38.94 ± 7.67	54.37 ± 7.67	51.76 ± 7.67	3.16	0.57
Serine	199.2 ± 18.1 *	227.5 ± 16.7	241.7 ± 18.1	268.5 ± 16.7 *	246.5 ± 16.7	254.2 ± 16.7	7.39	0.12
Glutamic acid	357.4 ± 42.5	314.8 ± 39.3	260.5 ± 42.5	284.7 ± 39.3	348.7 ± 39.3	340.9 ± 39.3	16.33	0.55
Glutamine	762.2 ± 54.4	674.4 ± 50.4	716.6 ± 54.4	664.1 ± 50.4	633.1 ± 50.4	653.8 ± 50.4	20.81	0.56
Glycine	1375 ± 143 ^a,#^	1556 ± 133 ^a^	1941 ± 143 ^ac,#^	2137 ± 133 ^c^	1832 ± 133 ^ac^	1899 ± 133 ^ac^	65.52	<0.01
Alanine	773.8 ± 84.3 ^a,#,^*	1111 ± 78.0 ^bc,^*	1103 ± 84.3 ^c,#^	1242 ± 78.0 ^bc^	1120 ± 78.0 ^bc^	1067 ± 78.0 ^ac^	37.73	0.01
Tyrosine	187.1 ± 8.94 ^a^	76.80 ± 8.27 ^b^	85.50 ± 8.94 ^b^	86.49 ± 8.27 ^b^	91.88 ± 8.27 ^b^	91.26 ± 8.27 ^b^	6.65	<0.01
Asparagine	160.0 ± 11.0 ^a^	100.0 ± 10.2 ^b^	110.0 ± 11.0 ^b^	109.5 ± 10.2 ^b^	101.4 ± 10.2 ^b^	97.38 ± 10.2 ^b^	5.19	<0.01
Proline	438.2 ± 29.7	364.2 ± 27.5	443.5 ± 29.7	430.3 ± 27.5	384.6 ± 27.5	406.3 ± 27.5	11.73	0.30
Cysteine	8.90 ± 2.30	8.10 ± 2.12	7.89 ± 2.30	6.15 ± 2.12	10.23 ± 2.12	9.18 ± 2.12	0.85	0.83
Ammonia	371.5 ± 59.3	404.8 ± 54.9	459.3 ± 59.3	455.2 ± 54.9	572.3 ± 54.9	494.5 ± 54.9	23.80	0.19
Creatinine	122.5 ± 12.4	153.2 ± 11.5	144.3 ± 12.4	164.2 ± 11.5	157.6 ± 11.5	163.0 ± 11.5	5.03	0.17
Taurine	156.3 ± 42.8	173.3 ± 39.6	136.5 ± 42.8 ^$^	272.3 ± 39.6	240.2 ± 39.6	310.0 ± 42.8 ^$^	18.65	0.03
Sarcosine	53.75 ± 5.89 ^a^	65.64 ± 5.45 ^ac^	65.75 ± 5.89 ^ac^	54.92 ± 5.45 ^a^	80.28 ± 5.4 ^bc^	67.58 ± 5.8 ^ac^	2.60	0.02
3,methylhistidine	9.50 ± 0.79	9.50 ± 0.73	8.16 ± 0.79	8.92 ± 0.79	8.92 ± 0.73	8.75 ± 0.79	0.30	0.83
1,methylhistidine	31.41 ± 3.82	22.78 ± 3.53	22.91 ± 3.82	28.71 ± 3.53	22.35 ± 3.53	18.25 ± 3.82	1.55	0.18
Ethanolamine	10.00 ± 2.11	13.14 ± 1.96	13.00 ± 2.11	14.64 ± 1.96	14.07 ± 1.96	11.75 ± 2.11	0.81	0.65
Carnosine	53.08 ± 13.2	29.00 ± 12.7	29.08 ± 13.2	36.21 ± 12.2	69.92 ± 12.2	22.50 ± 13.2	5.54	0.09
Hydroxyproline	140.2 ± 12.7	147.3 ± 11.7	143.0 ± 12.7	156.5 ± 11.7	145.9 ± 11.7	153.9 ± 11.7	4.68	0.92
Citrulline	66.65 ± 7.2	88.46 ± 6.7	80.80 ± 7.2	82.43 ± 6.7	80.14 ± 6.7	65.58 ± 6.75	2.96	0.14
Ornithine	213.0 ± 28.4	130.9 ± 26.2	129.9 ± 28.4	121.7 ± 26.2	168.8 ± 26.2	131.5 ± 26.2	11.42	0.18
α-amino butyric acid	30.25 ± 5.95	43.85 ± 5.51	41.25 ± 5.96	30.78 ± 5.51	42.35 ± 5.51	46.25 ± 5.95	2.40	0.24

^1^ PC (positive control): normal protein, adequate calcium (Ca) and available phosphorous (aP), no corn-expressed phytase (CEP) added; NC (negative control): low protein, adequate Ca and aP, no CEP added; LD: NC + CEP added at low dose, i.e., 2000 one phytase unit (FTU)/kg of diet; HD: NC + CEP added at high dose, i.e., 4000 FTU/kg of diet; LDR: LD with reduced Ca and P; HDR: HD with reduced Ca and P. Values are means ± SE. *n* = 6 for each dietary group. ^2^ SEM: Standard error of mean. ^a–c^ Within a row, values without a common superscript letter differ (*p* ≤ 0.05). *^,#,$^ Within a row, values with a common superscript symbol are tended to be different (0.05 < *p* ≤ 0.1).

**Table 3:** Proteins identified with significantly differential abundance in serum of nursery pigs fed with low-protein diets with or without reduced calcium and phosphorous supplemented with a corn-expressed phytase.

Protein ID	Protein Description	Molecular Function	Biological Process	Gene Name	*p*-Value
P21753	Actin monomer binding	actin monomer binding	regulation of cell migration	*TMSB10*	0.004
P68137	Actin, alpha skeletal muscle	ATP binding	gene expression regulation	*ACTA1*	0.000
Q95274	Thymosin beta 4 X-linked	actin monomer binding	regulation of cell migration	*TMSB4*	0.017
Q95JB4	Liver-expressed antimicrobial pept2	defense response to bacterium	-	*LEAP2*	0.041
F1SQ51	Sus scrofa basic proline-rich protein	-	-	*TP23*	0.044
Q9GKQ6	Uncharacterized protein	-	-	*BGN*	0.043
Q9TV62	Myosin, heavy chain 2, skeletal muscle	muscle contraction	actin filament binding	*MYH4*	0.001
A0A287A391	Eukaryotic translation elongation factor 1 alpha 1	GTPase activity	-	*EEF1A1*	0.031
A0A286ZYQ7	Ig-like domain-containing protein	-	-	*IGKV2D-40*	0.007
A0A287BLH9	Uncharacterized protein	-	immune response	*IGLV3-1*	0.023
A0A287A6F0	Myosin light chain, phosphorylatable, fast skeletal muscle	calcium ion binding	-	*MYLPF*	0.026
A0A287AAU1	Cellular communication network factor 3	insulin-like growth factor binding	signal transduction	*CCN3*	0.000
A0A287BAK1	Structural constituent of cytoskeleton	actin filament binding	cell adhesion	*TLN1*	0.024
A0A287B7U0	Ribosomal protein lateral stalk subunit P2	structural constituent of ribosome	cytoplasmic translational elongation	*RPLP2*	0.041
A0A5G2QYD1	Hyaluronic acid binding	carbohydrate binding	cell adhesion	*ACAN*	0.048
A0A5G2Q8S2	Peroxidasin	peroxidase activity	response to oxidative stress	*PXDN*	0.012
K7GRU7	Angiotensin-converting enzyme	carboxypeptidase activity	-	*ACE*	0.026
F1RVS9	Peptidase inhibitor 16	-	regulation of cell growth involved in cardiac muscle	*PI16*	0.000
F1SC51	Muscle alpha-actinin binding	actin binding	muscle structure development	*PDLIM1*	0.029
F1SFA7	Collagen type I alpha 2 chain	platelet-derived growth factor binding	bone mineralization	*COL1A2*	0.040
F1SKG1	Inhibin subunit beta E	cytokine activity	SMAD protein signal transduction	*INHBE*	0.046
I3L5L0	Tumor necrosis factor receptor superfamily member 10A-like	TRAIL binding	apoptotic process	*LOC100737977*	0.007
I3LDS3	keratin	protein heterodimerization activity	keratinocyte differentiation	*KRT10*	0.041
I3LM99	Grancalcin	calcium ion binding		*GCA*	0.001
T1UNN8	Angiopoietin like 8	-	regulation of protein processing	*ANGPTL8*	0.000

(-) not available in sus scrofa (pig) database: https://www.uniprot.org/ (accessed on 21 March 2021).

## Data Availability

Datasets supporting the results of this article are included within the article and its Supplementary Files.

## Supplementary Materials

The following supporting information can be downloaded at: https://www.mdpi.com/article/10.3390/ijms25010341/s1.
