# Supplementary material for: Recombinant Phytase Modulates Blood Amino Acids and Proteomics Profiles in Pigs Fed with Low-Protein, -Calcium, and -Phosphorous Diets
Source: Int J Mol Sci. 2023 Dec 26;25(1):341. doi: 10.3390/ijms25010341 (PMC10778770; doi:10.3390/ijms25010341)
Supplement: Supplementary file 1 [file ijms-25-00341-s001.zip › ijms-2757069-supplementary.pdf]

Supplementary Material

Supplementary Tables

Table S1. Paired-wise comparison of proteins identified with significantly differential abundance in serum of nursery pigs fed with low protein diets with or without reduced calcium and phosphorous supplemented with a corn-expressed phytase.

Protein ID	Protein description	Molecular function	Biological process	Gene name	*P*-value	Fold change
**NC vs PC[1]**						
T1UNN8	Angiopoietin like 8	structural constituent of cytoskeleton	cell adhesion	ANGPTL8	0.001	17.97
F6Q5X1	Serum amyloid A protein	Apolipoprotein of the HDL complex.	acute-phase response	SAA2	0.028	3.97
A0A287B0B8	Pulmonary surfactant-associated protein B	-	lipid metabolic process	SFTPB	0.003	2.91
A0A5K1TWC1	Apolipoprotein D	lipid binding	lipid transport	APOD	0.001	2.63
A0A5G2R684	Uncharacterized protein	-	-	MARCOL	0.018	2.54
Q28989	Ameloblastin	growth factor activity	biomineral tissue development	AMBN	0.033	2.52
A0A287ALJ6	Ig-like domain-containing protein	-	immune response	IGKV2D-29	0.011	2.37
I3LM99	Uncharacterized protein			GCA	0.000	2.24
A0A287AJ94	Microsemino protein, prostate associated	calcium ion binding	protein heterodimerization	MSMP	0.025	2.14
K7GM40	Apolipoprotein A1	cholesterol transfer activity	adrenal gland development	APOA1	0.031	2.08
F1RPX3	Secretoglobin family 1A member 1	-	signal transduction	SCGB1A1	0.042	1.97
F1RXM6	Serpin family A member 7	serine-type endopeptidase inhibitor activity	negative regulation of endopeptidase activity	SERPINA7	0.018	1.83
A0A287AEV6	Uncharacterized protein	-	positive regulation of neutrophil extravasation	CD99	0.015	1.83
A0A287AP28	Ig-like domain-containing protein	-	Glycoprotein	IGHV3-23	0.035	1.80
A0A5G2R113	Uncharacterized protein	dermokine	cornified envelope assembly	DMKN	0.026	1.73
I3L8B2	Uncharacterized protein	extracellular matrix structural constituent	extracellular matrix organization	COL9A2	0.040	1.70

A0A287APK0	6-phosphogluconolactonase (Pentose phosphate pathway)	6-phosphogluconolactonase activity	carbohydrate metabolic process	PGLS	0.050	1.61
P24853	Insulin like growth factor binding protein 2	insulin-like growth factor I binding	regulation of growth	IGFBP2	0.015	1.59
A0A5G2QE21	Uncharacterized protein	Serine peptidase inhibitor Kazal type 5	-	SPINK5	0.032	1.47
F1S6S9	Peptidase S1 domain-containing protein	Serine-type endopeptidase activity	negative regulation of phagocytosis	PRTN3	0.002	1.44
A0A5G2R4W0	LAM_G_DOMAIN domain-containing protein	Collagen type XVIII alpha 1 chain	-	COL18A1	0.038	1.37
A0A5G2QYD1	Aggrecan core protein	Hyaluronic acid binding	cell adhesion	ACAN	0.047	1.37
A0A287ASA4	Aa_trans domain-containing protein	Solute carrier family 38 member 10	-	SLC38A10	0.021	1.36
F1SFA7	Fibrillar collagen NC1 domain-containing protein	Platelet-derived growth factor binding	bone mineralization	COL1A2	0.033	-1.33
A0A2C9F3F9	Insulin like growth factor 1	growth factor activity	-	IGF1	0.017	-1.35
F1SM61	Fibulin 1	peptidase activator activity	extracellular matrix organization	FBLN1	0.043	-1.49
A0A5G2QJN1	LFNG O-fucosylpeptide 3-beta-N-Acetylglucosaminyltransferase.	metal ion binding	pattern specification process	LFNG	0.020	-1.62
F1RM86	Uncharacterized protein	Metalloendopeptidase activity	-	ADAMDEC1	0.030	-1.72
A0A287A391	Eukaryotic translation elongation factor 1 alpha 1	GTPase activity	-	EEF1A1	0.013	-2.00
A6P7L6	Calcitonin-related polypeptide beta	calcitonin receptor binding	regulation of cytosolic calcium ion concentration	CALCB	0.027	-2.42
I3L5L0	Uncharacterized protein	TRAIL binding	signal transduction	LOC100737977	0.037	-2.44
F1SCF1	SERPIN domain-containing protein	Serine-type endopeptidase inhibitor activity	negative regulation of endopeptidase activity	SERPINA6	0.029	-2.71
P00355	Glyceraldehyde-3-phosphate dehydrogenase (GAPDH)	peptidyl-cysteine S-nitrosylase activity	glucose metabolic process	GAPDH	0.022	-3.02
P68137	ATP binding (Actin)			ACTA1	0.001	-3.13
A0A287AAU1	Cellular communication network factor 3	insulin-like growth factor binding	-	CCN3	0.017	-3.19
F1RVS9	SCP domain-containing protein	Peptidase inhibitor 16	negative regulation of cell growth involved in cardiac muscle cell development	PI16	0.001	-3.60

A0A287BEI1	Keratin 77	-	-	KRT77	0.005	-4.38
F1RFS7	Matrix metallopeptidase 15	Cofactor for Ca and Zn	metalloendopeptidase activity	MMP15	0.032	-4.73
F1S8P9	Aldolase_II domain-containing protein	-	-	ADD1	0.042	-4.86
A0A5G2R4A8	Heterogeneous nuclear ribonucleoprotein A3	RNA binding	-	HNRNPA3	0.043	-5.18
A0A287ASI0	Keratin 7	-	-	KRT7	0.029	-5.55
A0A287A6F0	Uncharacterized protein	calcium ion binding	-	MYLPF	0.022	-10.86
Q9TV62	Myosin, heavy chain 2, skeletal muscle	actin filament binding	muscle contraction	MYH4	0.000	-14.40
**LD vs. NC**[1]						
I3LJP2	Selenoprotein P	selenium binding	regulation of growth	SELENOP	0.033	5.21
A0A5G2QPS7	Aldolase_II domain-containing protein	-	-	ADD2	0.050	4.33
A0A287BLH9	Uncharacterized protein	immunoglobulin production	-	IGLV3-1	0.022	3.63
A0A286ZTG2	Uncharacterized protein	metal ion binding	- Muscleblind like splicing regulator 1	MBNL1	0.015	2.32
F2Z5F7	Protein dpy-30 homolog isoform 1	protein homodimerization activity	endosomal transport	DPY30	0.026	2.29
A0A287B7U0	Ribosomal protein lateral stalk subunit P2	structural constituent of ribosome	translational elongation	RPLP2	0.022	2.23
F1S021	Collagen type V alpha 1 chain	platelet-derived growth factor binding	blood vessel development	COL5A1	0.024	1.83
A0A5G2QJN1	Beta-1,3-N-acetylglucosaminyltransferase	metal ion binding	pattern specification process	LFNG	0.007	1.66
A0A286ZL45	Dedicator of cytokinesis 2	guanyl-nucleotide exchange factor activity	actin cytoskeleton organization	DOCK2	0.048	1.41
A0A287ARV5	Ubiquitin related modifier 1	sulfur carrier	protein urmylation	URM1	0.024	1.34
A0A287AMT2	Uncharacterized protein	Laminin subunit beta 2	-	LAMB2	0.043	-1.33
Q9GKQ6	Biglycan	collagen fiber assembly	-	BGN	0.005	-1.49
F1RX36	Fibrinogen alpha chain	signaling receptor binding	plasminogen activation	FGA	0.037	-1.60

A0A287BGE8	Uncharacterized protein	-	-	GRN	0.038	-1.65
F1RJ72	BRICHOS domain-containing protein	-	endothelial cell morphogenesis	CNMD	0.045	-1.66
E9KYT3	Insulin like growth factor binding protein 4	insulin-like growth factor I binding	Either inhibit or stimulate the growth promoting effects of the IGFs	IGFBP4	0.039	-1.70
F1SCD0	SERPIN domain-containing protein	-	-	SERPINA3-2	0.017	-1.81
P06867	Plasminogen	serine-type endopeptidase activity	blood coagulation	PLG	0.035	-2.00
A0A5G2QWT2	Ig-like domain-containing protein	Glycoprotein	-	IGLV3-21	0.002	-2.06
A0A286ZYQ7	Ig-like domain-containing protein	Glycoprotein	-	IGKV2D-28	0.006	-2.80
A0A287ADT3	BCL2 like 15	-	regulation of apoptotic process	BCL2L15	0.041	-2.80
Q95JC8	Arginase 1, Catalytic activity	-	Involved in urea cycle	ARG1	0.048	-3.31
F1SCC7	SERPIN domain-containing protein	serine-type endopeptidase inhibitor activity	negative regulation of endopeptidase activity	LOC396684	0.004	-5.39
**HD vs. NC**[1]						
Q9TV62	Myosin, heavy chain 2, skeletal muscle	actin filament binding	muscle contraction	MYH1	0.004	20.38
F1SQ51	Uncharacterized protein	Basic proline-rich protein	-	TP23	0.015	17.30
A0A287B409	Uncharacterized protein	proton transmembrane transporter activity	transmembrane transport	IGKV2D-29	0.017	7.15
A0A287BEI1	IF rod domain-containing protein	-	-	KRT77	0.017	4.46
A0A5G2RFC8	Uncharacterized protein	-	-	UMAD1	0.043	3.83
F1RFS7	ZnMc domain-containing protein	zinc ion binding	-	MMP15	0.030	3.51
A0A5G2QW63	Uncharacterized protein	-	-	CCDC117	0.046	2.61
F1SR80	Tubulin alpha chain	GTPase activity	microtubule-based process	LOC100158003	0.043	2.01
A0A5G2RGV2	Integral membrane protein 2B	-	-	ITM2B	0.013	1.94
A0A5G2QJN1	Beta-1,3-N-acetylglucosaminyltransferase	O-fucosylpeptide 3-beta-N-acetylglucosaminyltransferase activity	pattern specification process	LFNG	0.036	1.87
A0A286ZL45	Uncharacterized protein	guanyl-nucleotide exchange factor activity	actin cytoskeleton organization	DOCK2	0.013	1.74

P16611	Insulin-like growth factor-binding protein 3	fibronectin binding	regulation of insulin-like growth factor receptor signaling pathway	IGFBP3	0.038	1.65
F1RM86	ADAM like decysin 1	metalloendopeptidase activity	-	ADAMDEC1	0.041	1.55
P01165	Neuroendocrine protein 7B2	unfolded protein binding	regulation of hormone secretion	SCG5	0.013	1.14
A0A287BGE8	Uncharacterized protein	-	-	GRN	0.026	-1.31
I3L5L0	Uncharacterized protein	TRAIL binding	signal transduction	LOC100624226	0.045	-1.63
I3LN42	Gc-globulin	calcidiol binding	vitamin D metabolic process	GC	0.017	-1.88
A0A287ALJ6	Ig-like domain-containing protein	immune response	-	IGKV2D-29	0.029	-2.12
F1SEC5	Cell growth regulator with EF-hand domain 1	calcium ion binding	-	CGREF1	0.003	-2.31
I3LS87	Transforming growth factor beta binding	transforming growth factor beta binding	cellular response to redox state	VASN	0.009	-2.49
A0A5G2QE94	Immunoglobulin superfamily containing leucine	-	-	ISLR	0.008	-2.61
A0A287B5C1	Uncharacterized protein	-	-	IGHM	0.008	-2.62
A0A287A0Y1	Uncharacterized protein	-	-	PFDN5	0.005	-4.40
A0A5G2RLN3	Calcium voltage-gated channel subunit alpha1 E	calcium ion binding	regulation of ion transmembrane transport	CACNA1E	0.036	-19.7
**LDR vs. LD[1]**						
I3LDS2	TMF1 regulated nuclear protein 1	DNA binding	regulation of cell cycle	TRNP1	0.014	11.68
A0A287ADT3	BCL2 like 15	regulation of apoptotic process	-	BCL2L15	0.022	6.43
Q95JC8	Arginase 1	Catalytic activity	involved in urea cycle	ARG1	0.028	6.11
I3LGN8	Plakophilin 1	cadherin binding	cell-cell junction assembly	PKP1	0.009	5.18
A0A287BHY5	keratin 2	cytoskeletal protein binding	intermediate filament organization	KRT2	0.024	5.02
I3LDS3	IF rod domain-containing protein	structural constituent of skin epidermis	peptide cross-linking	KRT10	0.016	4.91
A0A5G2QSE8	keratin 3, IF rod domain-containing protein	-	-	KRT3	0.026	4.42
F1SE02	Uncharacterized protein	-	-	CEP128	0.014	4.31
A0A287B8U7	Plexin domain containing 2	-	-	PLXDC2	0.046	3.22
A0A5G2RCI8	Coagulation factor XII	serine-type endopeptidase activity	-	F12	0.021	2.25
F1SHM0	Pyruvate kinase, muscle	pyruvate kinase activity	-	PKM	0.024	2.22

A0A286ZX29	serpin family B member 12	serine-type endopeptidase inhibitor activity	hematopoietic progenitor cell differentiation	SERPINB12	0.041	2.01
A0A287A1U5	Heat shock protein family E	ATP binding	protein folding	HSPE1	0.005	1.52
Q9GKQ6	Biglycan	Involved in collagen fiber assembly		BGN	0.025	1.40
I3LM99	Grancalcin	calcium ion binding	-	GCA	0.044	-1.41
P04119	Beta-lactoglobulin-1A/1C	retinol binding	-	LCN15	0.049	-1.50
A0A286ZTG2	Uncharacterized protein	Muscle blind like splicing regulator 1	-	MBNL1	0.005	-1.53
A0A287BRL8	Myristoylated alanine rich protein kinase C substrate	actin filament binding	actin filament bundle assembly	MARCKS	0.028	-1.79
I3LHP2	Acyl-protein thioesterase 2 isoform X3	lysophospholipase activity	protein depalmitoylation	LYPLA2	0.043	-1.83
A0A287B7U0	Ribosomal protein lateral stalk subunit P2	structural constituent of ribosome	translational elongation	RPLP2	0.024	-1.88
F1S862	Sulfiredoxin 1	sulfiredoxin activity	cellular response to oxidative stress	SRXN1	0.033	-2.02
I3LK29	Phosphatidylcholine-sterol acyltransferase	apolipoprotein A-I binding	cholesterol metabolic process	LCAT	0.018	-2.11
I3LEC2	Uncharacterized protein	mRNA binding	regulation of gene expression	PCBP1	0.021	-2.14
A0A5G2Q8S2	Peroxidasin	heme binding	response to oxidative stress	PXDN	0.014	-2.35
P21753	Thymosin beta-10	actin monomer binding	regulation of cell migration	TMSB10	0.013	-2.99
A0A287AAU1	Uncharacterized protein	insulin-like growth factor binding	-	CCN3	0.000	-3.19
**HDR vs. HD**[1]						
A0A5G2QXT5	Uncharacterized protein	-	-	IGHG4	0.020	2.36
F1SLW6	Uncharacterized protein	-	-	MFAP5	0.048	2.16
A0A287BRL8	Myristoylated alanine rich protein kinase C substrate	kinase activity	actin crosslink formation	MARCKS	0.037	1.56
A0A287BAK1	Talin 1	actin filament binding	cell adhesion	TLN1	0.049	-1.58
Q95274	Thymosin beta 4 X-linked	actin monomer binding	regulation of cell migration	TMSB4	0.004	-1.64
A0A287ACX2	Uncharacterized protein	centrosome localization	-	NIN	0.043	-1.66
A0A5G2R7T5	Uncharacterized protein	-	-	TMEM40	0.041	-1.80
P04574	Calpain small subunit 1	calcium ion binding	proteolysis	CAPNS1	0.021	-1.99
Q29290	Cystatin B	protease binding	adult locomotory behavior	CSTB	0.016	-1.99

A0A287ALA0	Brain abundant membrane attached signal protein 1	protein domain specific binding	glomerular visceral epithelial cell differentiation	BASP1	0.041	-2.15
P21753	Thymosin beta-10 (regulation of cell migration)	actin monomer binding	regulation of cell migration	TMSB10	0.014	-2.53
M3VJZ7	LIM and SH3 protein 1	actin filament binding	-	LASP1	0.000	-2.55
A0A5G2QRL2	PDZ domain-containing protein	-	-	PDLIM1	0.013	-2.62
K7GLE1	Annexin A1	phospholipase inhibitor activity	-	ANXA1	0.029	-2.71
F1RLQ3	Semaphorin 4A	semaphorin receptor binding	-	SEMA4A	0.027	-2.72
A0A287AKM8	Corneodesmosin	Protein homodimerization activity	skin morphogenesis	CDSN	0.047	-2.78
A0A287BEC7	Vesicle associated membrane protein 3	SNAP receptor activity	vesicle fusion	VAMP3	0.029	-3.10
A0A480PPD4	Eukaryotic translation initiation factor 4H	translation initiation factor activity	-	EIF4H	0.014	-3.33
A0A5G2QR43	Uncharacterized protein	-	-	GOLGB1	0.045	-3.39
P81693	Uncharacterized protein	-	-	ACP1	0.037	-3.77
A0A287BQI8	keratin, type II cytoskeletal 3-like	-	-	LOC100155249	0.030	-4.23
M3UZ42	IF rod domain-containing protein	brain development	Negative regulation of protein import into nucleus	UFM1	0.049	-7.38
A0A287B409	Uncharacterized protein	-	-	ATP6V0E1	0.004	-7.60

[1]PC (positive control): normal protein, adequate calcium (Ca) and available phosphorous (aP), no corn-expressed phytase (CEP) added; NC (negative control): low protein, adequate Ca and aP, no CEP added; LD: NC + CEP added at low dose, i.e. 2,000 one phytase unit (FTU)/kg of diet; HD: NC + CEP added at high dose, i.e. 4,000 FTU/kg of diet; LDR: LD with reduced Ca and P; HDR: HD with reduced Ca and P. n = 8 for each dietary group

[(-)] not available in sus scrofa (pig) database (https://www.uniprot.org/)

Supplementary Figures

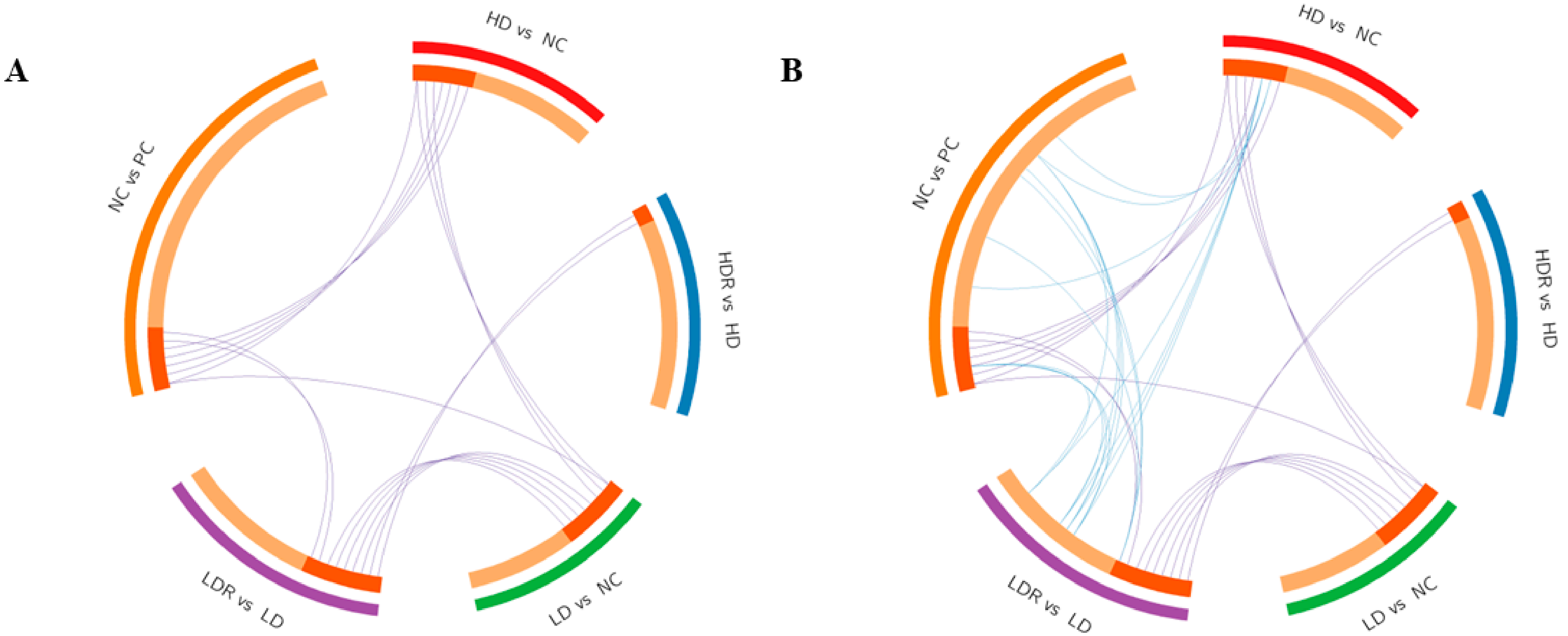

Figure S1. Overlap between gene lists: (A) at the gene level, where purple curves link identical genes; (B) including the shared term level, where blue curves link genes that belong to the same enriched ontology term. The inner circle represents gene lists, where hits are arranged along the arc. Genes that hit multiple lists are colored in dark orange, and genes unique to a list are shown in light orange. The greater the number of purple links and the longer the dark orange arcs imply greater overlap among the input gene lists. Blue links indicate the amount of functional overlap among the input gene lists. PC (positive control): normal protein, adequate calcium (Ca) and available phosphorous (aP), no corn-expressed phytase (CEP) added; NC (negative control): low protein, adequate Ca and aP, no CEP added; LD: NC + CEP added at low dose, *i.e.* 2,000 one phytase unit (FTU)/kg of diet; HD: NC + CEP added at high dose, *i.e.* 4,000 FTU/kg of diet; LDR: LD with reduced Ca and P; HDR: HD with reduced Ca and P. n = 7-8 for each dietary group.

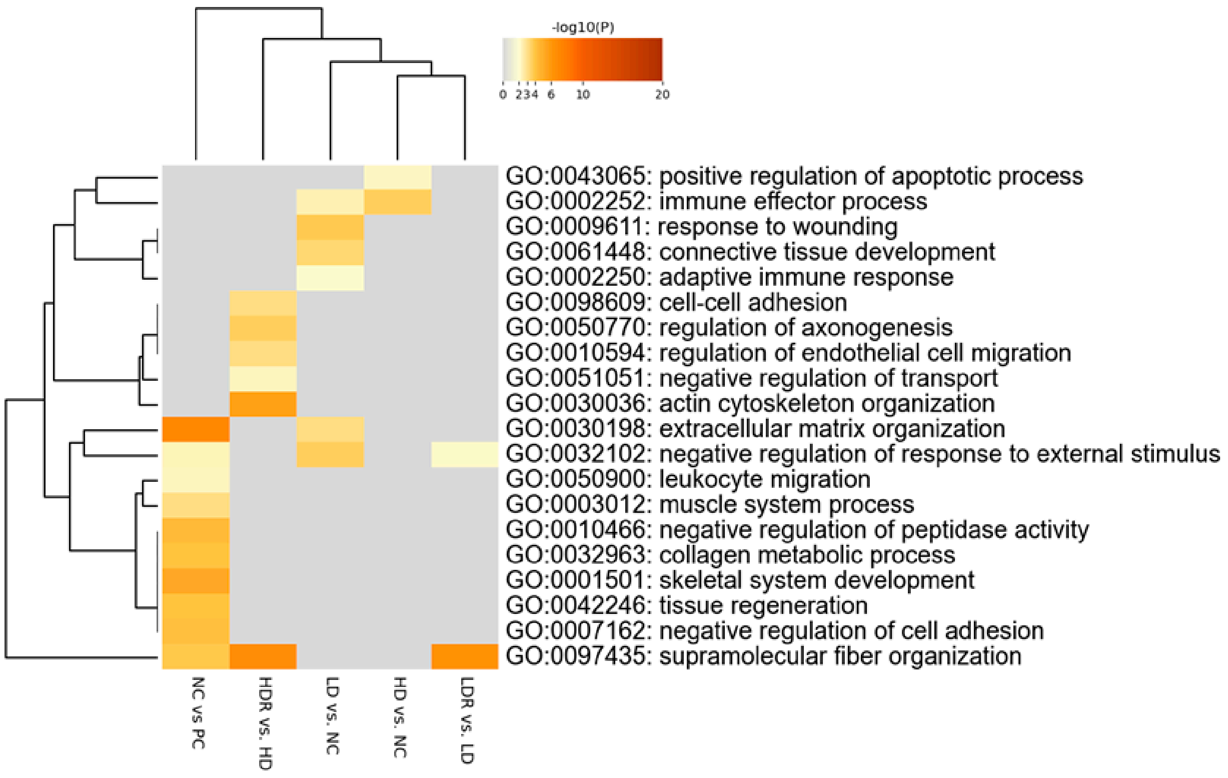

Figure S2. Overall biological processes enrichment analysis in Metascape. Significant terms are hierarchically clustered into a tree based on Kappa-statistical similarities among their gene memberships and a 0.3 kappa score was applied as the threshold to cast the tree into term clusters. Go term with the best P-value within each cluster was selected as its representative term and display them in a dendrogram. The heatmap cells are colored by their p-values, white cells indicate the lack of enrichment for that term in the corresponding gene list. PC (positive control): normal protein, adequate calcium (Ca) and available phosphorous (aP), no corn-expressed phytase (CEP) added; NC (negative control): low protein, adequate Ca and aP, no CEP added; LD: NC + CEP added at low dose, i.e. 2,000 one phytase unit (FTU)/kg of diet; HD: NC + CEP added at high dose, *i.e.* 4,000 FTU/kg of diet; LDR: LD with reduced Ca and P; HDR: HD with reduced Ca and P. n = 7-8 for each dietary group.

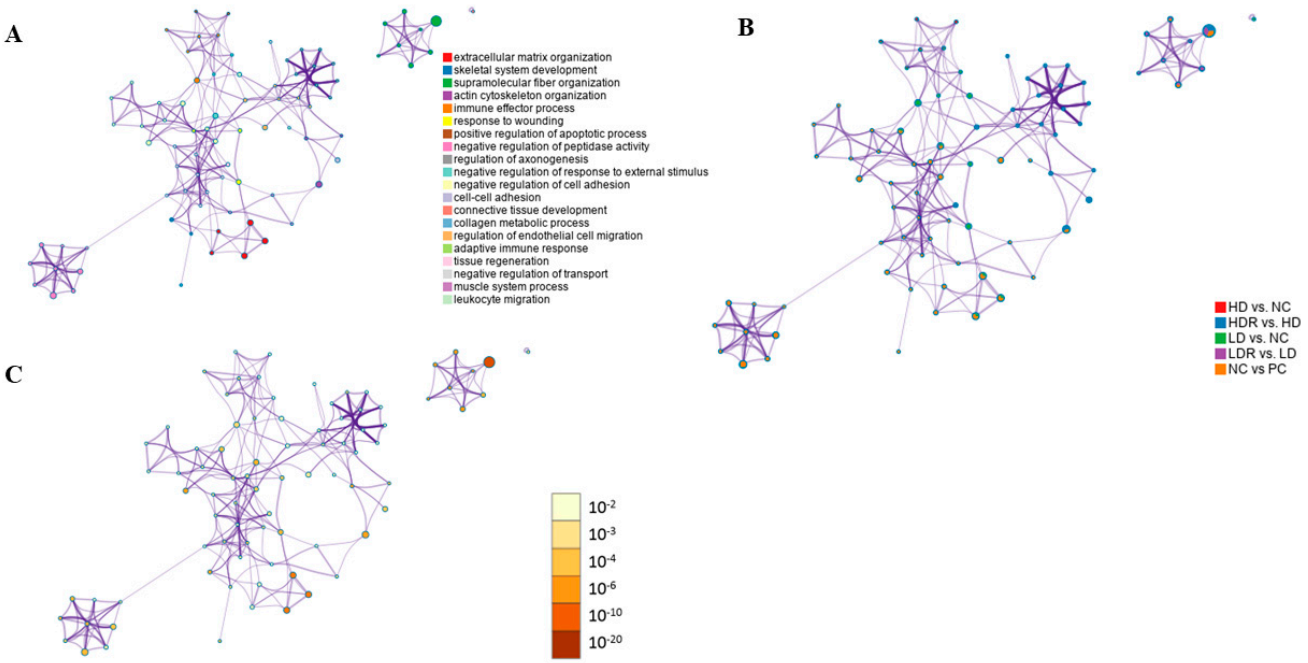

Figure S3. The selected subset of representative GO biological processes/terms from the full cluster were converted into a network layout. Terms with a similarity score > 0.3 are linked by an edge (the thickness of the edge represents the similarity score). One term from each cluster is selected to have its term description shown as label. (A) Enrichment network with nodes colored based on Go biological processes, (B) Enrichment network with nodes colored based on treatment groups, (C) Enrichment network with nodes colored by P-value. The darker the color the more the enrichment. PC (positive control): normal protein, adequate calcium (Ca) and available phosphorous (aP), no corn-expressed phytase (CEP) added; NC (negative control): low protein, adequate Ca and aP, no CEP added; LD: NC + CEP added at low dose, i.e. 2,000 one phytase unit (FTU)/kg of diet; HD: NC + CEP added at high dose, *i.e.* 4,000 FTU/kg of diet; LDR: LD with reduced Ca and P; HDR: HD with reduced Ca and P. n = 7-8 for each dietary group.

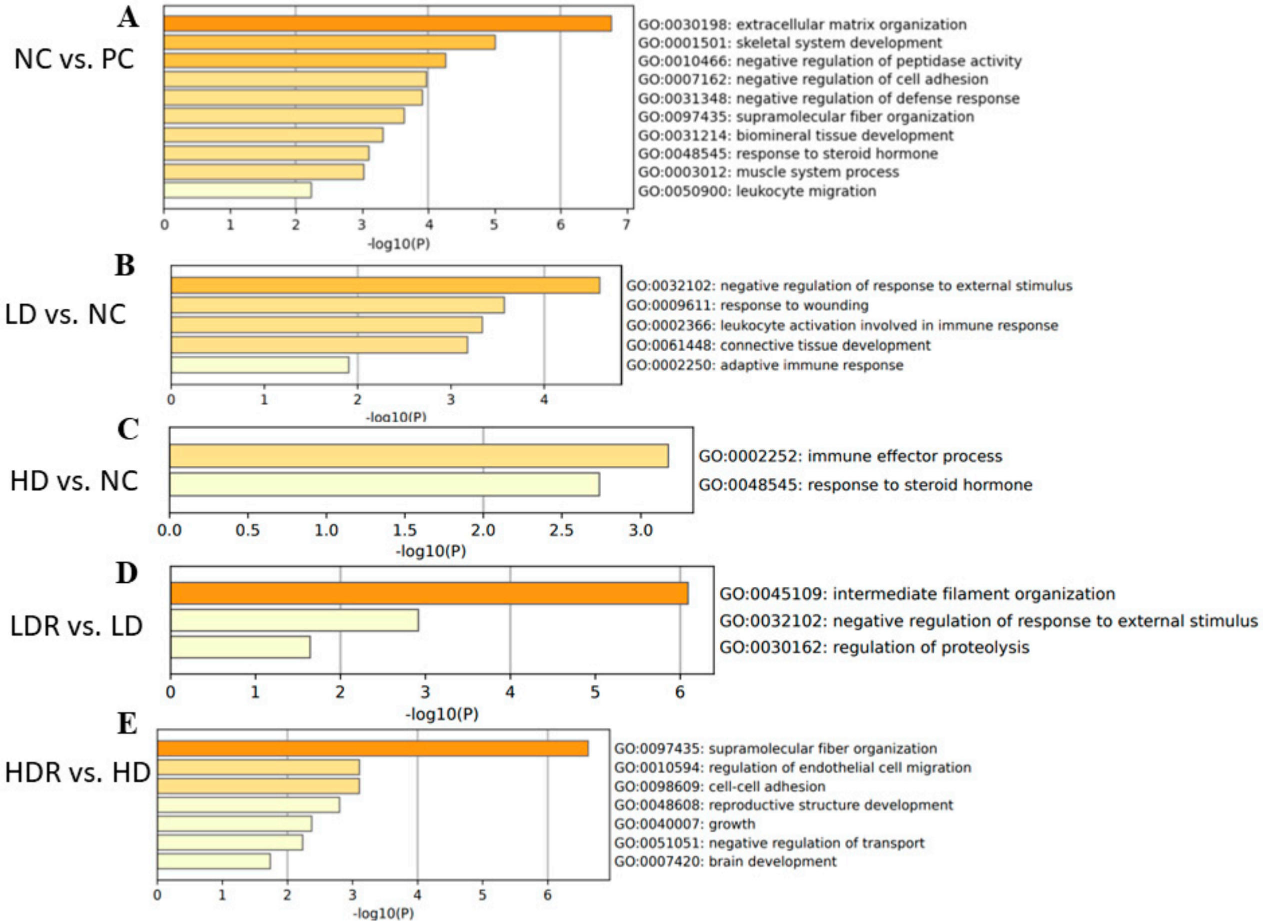

Figure S4. Gene enrichment GO analysis of differentially expressed proteins in serum of nursery pigs fed with low protein diets with or without reduced calcium and phosphorous supplemented with a corn-expressed phytase. PC (positive control): normal protein, adequate calcium (Ca) and available phosphorous (aP), no corn-expressed phytase (CEP) added; NC (negative control): low protein, adequate Ca and aP, no CEP added; LD: NC + CEP added at low dose*, i.e.* 2,000 one phytase unit (FTU)/kg of diet; HD: NC + CEP added at high dose, *i.e.* 4,000 FTU/kg of diet; LDR: LD with reduced Ca and P; HDR: HD with reduced Ca and P. n = 7-8 for each dietary group.
